# Gastric mucosal pathology in Belgian Shepherd dogs with and without clinical signs of gastric disease

**DOI:** 10.1186/s13028-021-00570-6

**Published:** 2021-02-09

**Authors:** Marcus Vinicius Cândido, Pernillä Syrjä, Mohsen Hanifeh, Jaan Lepajõe, Kati Salla, Susanne Kilpinen, Peter-John Mäntylä Noble, Thomas Spillmann

**Affiliations:** 1 Department of Equine and Small Animal Medicine, Faculty of Veterinary Medicine, University of Helsinki, P.O. Box 57, 00014 Helsinki, Finland; 2 Department of Veterinary Biosciences, Faculty of Veterinary Medicine, University of Helsinki, P.O. Box 66, 00014 Helsinki, Finland; 3 Institute of Veterinary Science, Faculty of Veterinary Medicine, University of Liverpool, Chester High Road, NestonWirral, CH64 7TE UK

**Keywords:** Atrophy, Biopsy, Carcinoma, Dysplasia, Endoscopy, Metaplasia

## Abstract

**Background:**

Gastric carcinoma (GC) is uncommon in dogs, except in predisposed breeds such as Belgian Shepherd dogs (BSD) of the Tervuren and Groenendael varieties. When GC is diagnosed in dogs it is often late in the disease, resulting in a poorer prognosis. The aim of this prospective clinical study was to investigate possible associations of gastric mucosal pathologies with clinical signs, laboratory test results and GC in BSD. An online survey gathered epidemiological data to generate potential risk factors for vomiting as the predominant gastric clinical sign, and supported patient recruitment for endoscopy. Canine Chronic Enteropathy Clinical Activity Index (CCECAI) score and signs of gastroesophageal reflux (GER) were used to allocate BSD older than five years to either Group A, with signs of gastric disease, or Group B, without signs. Findings in the clinical history, laboratory tests and gastric histopathology of endoscopic biopsies were statistically analysed in search of associations.

**Results:**

The online survey included 232 responses. Logistic regression analysis recognized an association of vomiting with gagging, poor appetite and change in attitude. Recruitment for endoscopy included 16 BSD in Group A (mean age 9.1 ± 1.8 years, mean CCECAI = 3.1 ± 2.2 and signs of GER); and 11 in Group B (mean age 9.8 ± 1.4 years, CCECAI = 0, no signs of GER). Seven (25.9%) of the 27 BSD (Group A 4/16, Group B 3/11) had leukopenia. Serum C-reactive protein tended to be increased with more advanced GC (P = 0.063). Frequency of GC, mucosal atrophy, mucous metaplasia, or glandular dysplasia did not differ between groups. GC was frequently diagnosed (6/27), even without clinical signs (2/11). The odds ratio for vomiting (OR = 9.9; P = 0.016) was increased only when glandular dysplasia was present. GC was associated with mucous metaplasia (P = 0.024) and glandular dysplasia (P = 0.006), but not with mucosal atrophy (P = 1).

**Conclusions:**

GC can develop as an occult disease, associated with metaplasia and dysplasia of the gastric mucosa. Suggestive clinical signs, notably vomiting, should warrant timely endoscopy in BSD. Extensive endoscopic screening of asymptomatic dogs remains, however, unrealistic. Therefore, biomarkers of mucosal pathology preceding clinical illness are needed to support an indication for endoscopy and enable early diagnosis of GC.

## Background

Gastric carcinoma (GC), the most common gastric neoplasm in dogs, is relatively rare, representing less than 1% of canine cancers. However, a few lineages within certain dog breeds are predisposed to GC. GC is frequently seen in Tervuren and Groenendael Belgian Shepherd dogs (BSD) and some other dog breeds, including Bouvier des Flandres, Collie, Standard Poodle, Norwegian Elkhound and Chow-Chow [1–5]. A previous study in Finland found an increased risk ratio (RR = 19) for Tervuren BSD undergoing endoscopy to be diagnosed with GC [6]. In the majority of cases, GC carries a very poor prognosis, since diagnosis is mostly achieved at a late, advanced stage. The median age at diagnosis ranges from 8 to 10 years, but occasional cases have been reported in dogs younger than 5 years [4, 7, 8]. Standard white light endoscopy (WLE) with mucosal sampling is conventionally used to support the diagnosis, but sometimes full-thickness gastric biopsy is required [4].

In dogs with GC, non-specific clinical signs of upper gastrointestinal (GI) illness tend to appear late in the course of disease, at a time point when surgical resection is seldom curative since the tumour has already metastasised [4, 7, 9]. In contrast, early diagnosis and resection can improve patient survival and welfare. A recent retrospective case series reported a median survival time of just 178 days after surgical treatment in 40 dogs with GC [9]. However, such survival time was about twice as long as those reported for non-operated patients [4, 7, 10]. Moreover, 7/40 dogs survived longer than one year after surgical resection with prolonged survival times of up to 1443 days [9].

In humans, early diagnosis of gastric cancer is an important factor for long-term survival, and endoscopy with gastric mucosal biopsy is the most valuable diagnostic tool [11]. However, gastric mucosal pathology including gastric mucous or intestinal metaplasia and dysplasia can present as flat lesions that are easily overlooked when using WLE alone [11–13]. In human medicine, metaplastic and dysplastic changes of the gastric mucosa are regarded as preneoplastic and demand systematic staging [14]. Therefore, new techniques such as chromoendoscopy and narrow band imaging have been increasingly applied since they allow for better visualization and sampling of gastric mucosal pathology [11, 15, 16]. In veterinary medicine, early endoscopic diagnosis of canine GC and its association with mucosal changes regarded as preneoplastic have yet to be investigated. Furthermore, in contrast to human medicine, protocols for staging GC are still missing.

Studies on clinical presentation and early diagnosis of GC in dogs remain scarce. Clinical signs consistently reported in association with GC are vomiting, anorexia and weight loss, but many other signs such as ptyalism, gagging, retching, apathy, lethargy and cachexia, and occasionally melaena and abdominal pain, can be present [2, 4, 7, 10]. Vomiting is the predominant sign associated with gastric disease, affecting 53.7% of the dogs with gastric histopathological abnormalities [17]. Appetite loss is a common feature of GC [4, 7], but a ravenous appetite in spite of weight loss has also been reported [9]. Body condition score tends to be reduced in dogs with GC as compared to those with gastritis and control dogs [18]. Non-specific clinical signs of upper gastrointestinal disease may also be related to gastroesophageal reflux (GER). GER may cause upper GI signs such as repetitive lip smacking, increased empty swallowing motions, chronic intermittent vomiting, sudden unexplained discomfort, belching, drooling, excessive grass eating, presumed postprandial pain, refusal to eat despite interest, regurgitation, retching, excessive surface licking etc. Most of these clinical signs of upper GI disease are not specific and may also refer to gastritis or chronic inflammatory enteropathies [19].

There is conflicting information about the role of grass eating in upper GI diseases. In veterinary textbooks, this non-specific sign is sometimes considered a possible mechanism for self-induced vomiting in nauseated animals [20]. In contrast, fructo-oligosaccharide supplementation to induce mild GI disturbance in dogs did not lead to using grass as an emetic [21]. Other authors concluded that grass-eating should be regarded as a normal behaviour of dogs [22–24]. Excessive surface or object licking was found to be a behavioural change associated with gastrointestinal abnormalities [25]. Currently it is unknown to which extent such non-specific signs are associated with early or advanced gastric mucosal pathology in dogs.

Blood test abnormalities may be absent in dogs with GC or tend to be minor, and may include mild anaemia (microcytic, hypochromic), hypoglycaemia, hyperproteinaemia, hypoalbuminaemia and increased liver values [4, 7, 10]. Moreover, dogs with GC can present with increased levels of serum C-reactive protein (CRP) and decreased serum folate concentrations as compared to dogs with chronic gastritis and healthy controls [18]. A previous retrospective study in dogs with gastric histopathological abnormalities found that 44% of dogs had lymphopenia and 33% had neutrophilia, whereas 14% had lymphocytosis [17].

This prospective study is a first attempt to improve the early diagnosis of canine GC by comparing clinical history with endoscopic and histological findings of gastric mucosal biopsies in Tervuren and Groenendael BSD with and without clinical signs of upper GI disease.

The main objective of the study was to investigate possible associations of gastric mucosal pathology with signalment, clinical signs (specific and non-specific signs of upper GI disease) and selected standard laboratory tests (basic haematology and serum biochemistry; CRP).

The secondary objective was to test for possible associations between gastric histolopathogical changes and GC, and their potential involvement as preneoplastic changes in dogs.

## Methods

### Belgian Shepherd dog population survey

An electronic questionnaire was available at the University’s canine research webpage, and it was advertised along with links in the website of the Belgian Shepherd Dog Association of Finland. The data supplied an epidemiologic survey and facilitated the recruitment of dogs to the prospective study. Only complete responses concerning pedigree-registered dogs were considered. The enquiry included:

- Breed variety (Tervuren, Groenendael, Malinois, Lakenois);
- Age and sex;
- Feeding (number of meals per day) and diet (commercial processed or raw food, treats);
- Incidence and frequency of vomiting (whether after eating or on empty stomach);
- Presence of gagging;
- Presence of ‘altered appetite’ including grass-eating, licking surfaces or objects, eating foreign material such as soil, faeces, wood, toys etc. (pica);
- Weight loss;
- Being regarded as nervous, or depressed, or with a change in behaviour or disposition;
- Previous clinical abnormalities (blood tests, chronic disease);
- Current medication.

### Patient selection for endoscopic examinations

Survey responses facilitated patient recruitment. The owners of BSD older than 5 years were actively contacted following the chronological order of response to the survey, to minimize potential bias, e.g. related to individual interest in the outcomes of the examinations or of the research. Volunteers were invited for a clinical visit. During anamnesis, the owners were specifically questioned about gastrointestinal signs such as vomiting (including frequency), and signs of gastroesophageal reflux. Dogs underwent physical examination and samples were taken for routine laboratory tests including complete blood count (CBC), serum biochemistry and faecal parasitology. The resulting information was used for patient inclusion and grouping.

#### Inclusion criteria

Individual dogs were included in accordance with prospectively defined criteria (breed type: Tervuren or Groenendael, age above five years). Patient grouping was based on the Canine Chronic Enteropathy Clinical Activity Index (CCECAI), an indicator primarily targeted for enteropathies that also includes criteria referable to gastric illness such as patient behaviour, appetite, weight loss, and vomiting [26]. Along with CCECAI score, signs suggestive of GER such as gagging, retching, belching (eructation), lip smacking or licking and repetitive swallowing [19] were used to assign the dogs to mutually exclusive groups: with or without clinical signs referable to gastric disease. Group A consisted of dogs with CCECAI score ≥ 1 and/or presence of signs of GER. Group B comprised dogs with CCECAI = 0 and no signs of GER.

#### Exclusion criteria

Malinois and Lakenois BSD were excluded from the clinical study due to the lack of evidence for predisposition to GC in these breeds [1]. Dogs previously diagnosed with concurrent or chronic non-gastic disease, or regarded at an increased anaesthetic risk, were also excluded.

### Laboratory work

Blood was collected in EDTA tubes for CBC including differential white blood cell count (WBC) and in serum tubes for biochemistry, including CRP. The resulting information was used to support patient inclusion, grouping and further statistical analysis. Remaining EDTA-blood and serum, as well as faecal samples, were frozen at − 20 °C for 6–18 months and then transferred to storage at − 80 °C. Serum CRP was measured 10 months later as a single batch, applying a particle-enhanced turbidimetric immunoassay (Gentian Diagnostics AS, Norway) analyzed using Konelab 60i (Thermo Fisher Scientific, USA).

### Endoscopic equipment and procedures

Dogs of Groups A and B underwent a clinical study based on gastroscopy (endoscope CF180AL, Olympus Europe) with mucosal biopsy sampling (disposable forceps FB-210U, Olympus Europe). The procedures were performed under anaesthesia as described elsewhere [27].

**Fig. 1:**
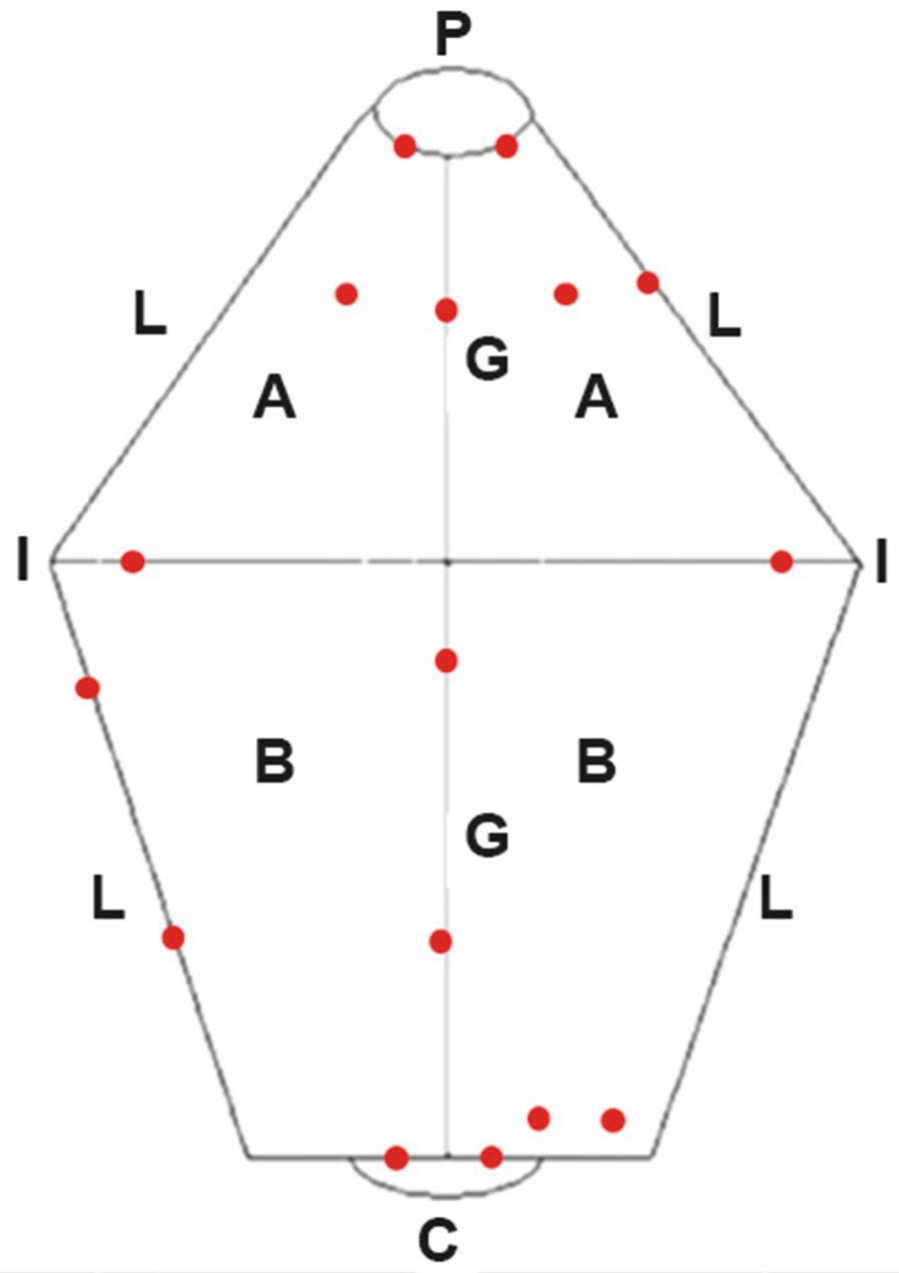
Illustration of the stomach showing standard biopsy sites (red dots) used for non-targeted gastric mucosal sampling ( modified from Simone et al. [30]). A: antrum; B: gastric body; G: greater curvature; L: lesser curvature; P: pylorus; I: incisura angularis (angular fold); C: cardia

Biopsy site selection for focal lesions was facilitated by image enhancement using two subsequent techniques: 1) narrow band imaging (NBI) [28] and chromoendoscopy [16]. NBI was enabled by a specific light source (CLV-180, Olympus Europe) and image processor (CV-180, Olympus Europe) to enhance mucosal and vascular assessment [29]. For chromoendoscopy, 0.2% indigo-carmine was applied to the mucosal surface with an endoscopic spray catheter (PW-5 V-1 Olympus Europe) to improve mucosal topographic assessment [16]. A schematic gastric map was used to record visible changes [30]. Visible lesions from all gastric regions were sampled. In the absence of visible lesions, sampling from predefined positions in each gastric region was undertaken, namely two samples each from cardia, fundus, greater and lesser curvature of gastric body, incisura angularis and pylorus, and one from each quadrant of the antrum (clockwise at 12, 3, 6 and 9 o’clock) (Fig. 1). An annual follow-up endoscopy was offered for dogs diagnosed with dysplastic lesions.

### Histologic staining and histopathological assessment

The mucosal samples were placed onto wood chips then immersed in 10% formalin for fixation, using separate vials labelled in reference to the respective gastric region (cardia and fundus; gastric body; incisura; antrum and pylorus), or focal lesion (e.g. ulcer; mass; texture change). The biopsies were paraffin-embedded, cut into four-micrometre-thick sections and stained with haematoxylin–eosin. Special staining techniques (periodic acid-Schiff reaction) were applied at the pathologist’s discretion. Immuno-histochemical staining for epithelial cells was performed using an anti-cytokeratin AE1/AE antibody (anti-human Ck AE1/AE3, mouse monoclonal M3515, Dako Agilent, USA). The antigens were retrieved with 0.01 M citrate buffer at pH 6 and heated for 20 min at 99 °C. The signal was revealed according to instructions for the UltraVision Detection System HRP/DAB kit (Thermo Fisher Scientific, USA).

Histological slides were examined by a board-certified pathologist following the template recommended by the World Small Animal Veterinary Association’s International Gastrointestinal Standardization Group [31]. Mucous metaplasia, glandular dysplasia and GC type were recorded [32, 33]. Mucosal inflammation, i.e. intraepithelial lymphocytes, and lymphoplasmacytic, eosinophilic and neutrophilic infiltration into lamina propria, was scored as normal = 0, mild = 1, moderate = 2 or 3 = severe [34]. An individual mean severity score (MSS) of gastric mucosal inflammation was calculated from the average of those four scores in each dog, for statistical purposes.

### Statistical methods

For data analysis of the epidemiological survey, signalment, feeding, and clinical and behavioural variables were treated as potential risk factors for vomiting, the most relevant clinical sign for gastric localization. For that purpose, a cut-off for vomiting was set at a frequency of ≥ 1 episode per month. The factors were first assessed separately with univariate logistic regression. Factors deemed significant underwent a multivariate model, where main effects were simultaneously included as fixed effects.

Concerning data analysis in the endoscopic clinical study, the updated clinical information of patients was compared with findings from endoscopy and histopathology, including the calculated MSS of gastric mucosal inflammation. D’Agostino & Pearson’s test was applied for normality of distribution (confirmed when α < 0.05); Welch’s *t*-test was applied in search of sex differences, both within groups and independently of grouping, concerning CCECAI score (Group A), WBC, CRP, and MSS, using Prism 8 for Windows (GraphPad Software, USA). Presence of gastric mucosal atrophy, metaplasia, and dysplasia as well as GC were treated as binary variables. The effects of vomiting, GER and Group on histopathological variables were analysed by logistic regression. The same applied for the effects of atrophy, metaplasia, dysplasia and GC on the incidence of vomiting and serum CRP concentration, as well as the effect of vomiting on CRP. Odds ratios (OR) with 95% confidence intervals (CI) were calculated to quantify the results of the logistic regression analyses. The effects of clinical signs and histopathological variables on MSS were analysed by analysis of variance and tests of fixed effects; a calculated Pr > F value < 0.05 was deemed significant. The possible association between GC and gastric mucosal atrophy, mucous metaplasia and glandular dysplasia was tested with Fisher’s exact tests. P-values < 0.05 were considered statistically significant. Except otherwise noted, SAS System for Windows, version 9.4 (SAS Institute Inc., USA) was used for statistical analyses.

## Results

### Belgian Shepherd dog population survey

Out of 232 valid answers to the survey, 120 BSD were female (51.7%) and 112 were male (48.3%); 98 were Tervuren (42.2%), 64 Groenendael (27.6%), 65 Malinois (28%) and five Lakenois (2.2%). The mean age was 6.9 ± 3.4 years. Age followed a normal distribution.

**Fig. 2:**
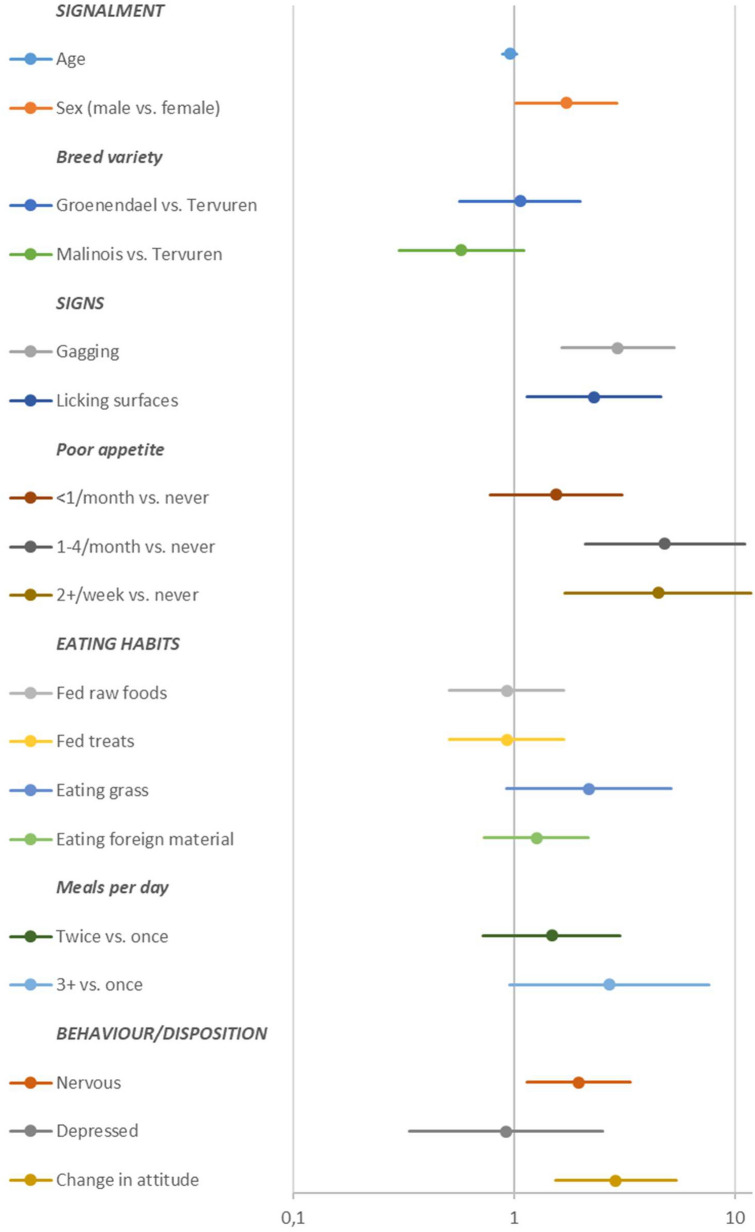
Forest plot of odds ratios for vomiting from the univariate analyses of signalment, clinical signs, feeding and behaviour

Eating history revealed that about 88% of the dogs were fed treats, 87.5% were eating grass and 34% were eating foreign material. Vomiting once a month or more often was reported in 43% of the dogs, gagging in 29% and excessive licking of surfaces in 17%. The univariate logistic regression (Fig. 2) indicated an association of vomiting with male sex (P = 0.04), licking surfaces (P = 0.02) and gagging (P < 0.001), as well as poor appetite reported once a month or more often (P < 0.002), reported change in attitude or behaviour (P = 0.001) and being regarded as nervous (P = 0.015). Breed variety showed no significance as a risk factor for vomiting when Tervuren was compared to Groenendael (P = 0.85) or Malinois (P = 0.1).

Concerning the number of meals, dogs fed once a day (18%) apparently vomited less often than those fed more often or ad libitum (82%), but significance was unproven when “once a day” was compared to “three times a day or more often” (P = 0.06) (Fig. 2). Vomiting on an empty stomach was reported in 85% of dogs fed once a day and in 73% of dogs fed more often. Other predefined risk factors, such as patient age, eating treats, eating grass, eating foreign material, being fed raw food or being regarded as depressed, failed to show statistical effect on vomiting (Fig. 2).

**Fig. 3:**
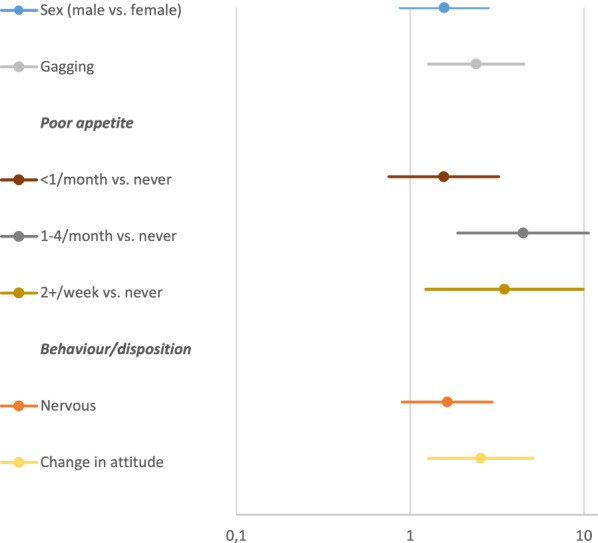
Forest plot of odds ratios for vomiting from the multivariate analyses of signalment, clinical signs and behaviour

Although the univariate model suggested vomiting to occur twice as often in males than in females, sex failed significance as a risk factor for vomiting in the multivariate analysis (P = 0.13) (Figs. 2, 3). The multivariate model also contradicted the association of vomiting with sex of the dog (P = 0.14), licking objects or surfaces (P = 0.28), or being regarded as nervous (P = 0.13). The association with vomiting was confirmed for the following factors: gagging (P = 0.02), change in behaviour or attitude (P = 0.01), and refusal to eat, both occurring once a month to once a week (P < 0.001) and more often than twice a week (P = 0.018) (Fig. 3).

### Endoscopic clinical study

#### Patient inclusion and grouping

Based on the responses to the survey, 79 dogs younger than 5 years of age were initially excluded as the disease is known to affect older dogs. Thirty-five owners of BSDs with age >5 years volunteered to bring their dogs for assessment. However, five dogs were Malinois and were not included in the study. Two male Groenendaels were excluded due to concurrent chronic disease such as spondylosis, recurrent urinary tract infections and borreliosis diagnosed one year earlier. An asymptomatic, severely obese female Tervuren was excluded on account of increased anaesthetic risk.

**Fig. 4:**
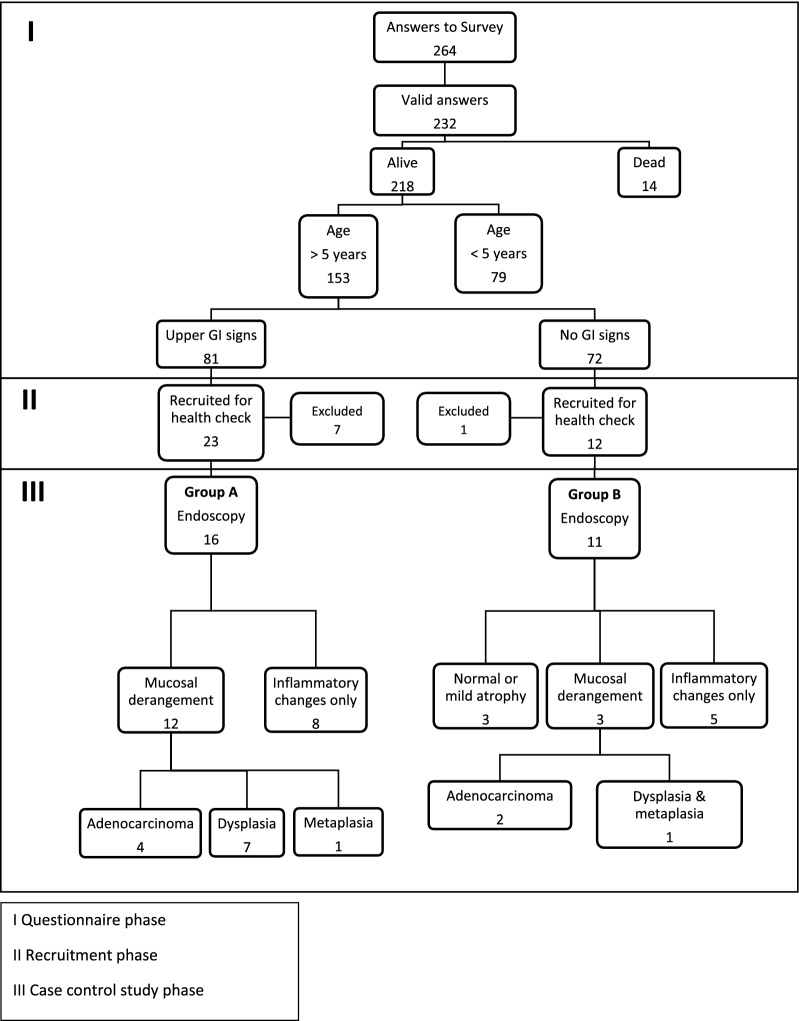
Flowchart showing patient recruitment, inclusion, most severe diagnosis and outcome of affected dogs. During the first phase, the answers to the survey allowed to contact owners of potential study patients. The second phase involved clinical assessment including updated anamnesis, physical examination, blood and faecal sampling. In the third phase, dogs included in the study were scoped, with biopsy sampling leading to final histopathological diagnosis

A total of 27 BSD (19 Tervuren, eight Groenendael) with a mean age of 9.4 ± 1.7 years underwent gastroscopy. Age followed a normal distribution both considering all dogs included and each of the study Groups A and B (Fig. 4). Individual patient information is summarized in Table 1.

**Table 1:** Belgian Shepherd dogs with (Group A) or without upper gastrointestinal clinical signs (Group B) included in endoscopic study: clinical presentation, blood parameters and histopathological changes

Patient no	Breed type	Age	Clinical presentation		Blood work		Histology	
Dog, sex			CCECAI score	GER	WBC	CRP	Findings	MSS
*Group A*								
642, M	Tervuren	9.9	6	–	14.8	29.1		1
644, F*n*	Tervuren	10.1	2	–	6.6	8.8	A	2
645, F*n*	Tervuren	11.6	3	–	7.2	14.7		2
646, M	Groenendael	11.2	3	–	5.6	9.6	A, M, D	1
647, F*n*	Tervuren	10.1	1	–	6.3	15.3	M, D, U-GC	2
649, F	Tervuren	7.9	2	–	6.3	8.2	A	2
650, M	Groenendael	10.6	3	–	6.5	7.4	A	2
652, M*n*	Tervuren	10.1	1	–	3.8	10.7	D	2
655, F*n*	Groenendael	9.1	3	Yes	5.4	10.5	A	1
658, M	Tervuren	7.2	8	–	8.0	15.5	A, M, D, U-GC	2
664, F*n*	Tervuren	10.4	1	–	5.1	5.9	A, D	2
677, F	Groenendael	8.3	6	Yes	5.4	24.4	A, D, U-GC	2
680, F	Tervuren	5.5	5	Yes	6.6	5.3	M, D	1
686, F	Tervuren	5.6	2	Yes	5.5	45.1	A, D	2
687, F*n*	Groenendael	9.7	4	Yes	5.3	10.0	D	3
711, F*n*	Groenendael	8.3	0	Yes	4.9	11.0	A, D, GC	2
*Group B*								
641, M	Tervuren	10.8	0	–	9.7	9.4	A, M, D, GC	2
679, F*n*	Tervuren	9.0	0	–	4.0	12.1	N	2
703, M	Tervuren	9.0	0	–	7.8	8.5	A	2
719, F*n*	Tervuren	11.3	0	–	5.6	12.0	A	1
721, F*n*	Tervuren	11.2	0	–	5.3	13.5	A, M, D, U-GC	1
722, M	Tervuren	11.2	0	–	7.6	6.5	A, M,D	3
723, M	Tervuren	11.0	0	–	7.0	6.8	A	1
724, F	Groenendael	7.6	0	–	4.9	9.4	A	1
747, F	Tervuren	8.2	0	–	5.4	8.7	A	1
749, M*n*	Groenendael	8.3	0	–	5.6	8.3	A	0
750, F*n*	Tervuren	10.1	0	–	5.6	8.4	A	2

Age in years. M: male; F: female; *n*: neutered, CCECAI: canine chronic enteropathy clinical activity index, GER: signs referable to gastroesophageal reflux (gagging, eructation, smacking, licking lips and swallowing), WBC: total leucocyte count (reference range: 5.4–17.4 × 10^9^/L), CRP: C-reactive protein in serum (reference value: < 10 mg/L), A: gastric mucosal atrophy; M: mucous metaplasia; D: glandular dysplasia; U-: ulcerated; GC: gastric carcinoma, N: normal histology, absence of pathological changes, MSS: mean severity score (0 = normal; 1 = mild; 2 = moderate; 3 = severe); average of four histopathological scores [31]: intraepithelial lymphocyte count and inflammatory infiltrations in lamina propria (lymphoplasmacytic, eosinophilic and neutrophilic)

*Group A* included 16 BSD with signs of gastric disease (10 Tervuren: three males, two intact and one castrasted, and seven females, three intact and four spayed; six Groenendael: two intact males, one intact and three spayed females). Mean age was 9.1 ± 1.8 years. Mean CCECAI score was 3.1 ± 2.2. All 16 dogs were vomiting once a month or more often and six of them also showed signs of GER. CCECAI score followed a normal distribution within Group A. There was no significant difference between males (mean CCECAI = 4.2 ± 2.8) and females (mean CCECAI = 2.6 ± 1.8) (P = 0.3).

*Group B* included 11 BSD without signs of gastric disease (nine Tervuren: four intact males, one intact and four spayed females; two Groenendael: one castrated male and one intact female). Mean age was 9.8 ± 1.4 years. All of these dogs had CCECAI score = 0 and no signs referable to GER.

All BSD in the endoscopic study were reported to eat grass, except for one (dog 641, Group B).

#### Laboratory findings

Median WBC in Group A was 5.9 × 10^9^/L (range 3.8–14.8) and in Group B, 5.6 × 10^9^/L (range 4–9.7); the difference was non-significant (P = 0.77). WBC followed a normal distribution within Group B (mean WBC = 6.2 ± 1.6), but not in Group A. WBC did not differ between males and females (P = 0.13) with or without regard to grouping. Without regard to grouping, median WBC for all dogs was 5.6 × 10^9^/L (range 3.8–14.8) and seven (25.9%) of the 27 dogs (Group A 4/16, Group B 3/11) had WBC below the lower limit of reference range (5.4–17.4 × 10^9^/L). Two dogs in each group (12.9%) had absolute neutropenia (minimum 2 × 10^9^ neutrophils/L; reference 2.9–13.8 × 10^9^/L), one of which had also lymphopenia (dog 652; Group A; 0.89 × 10^9^ lymphocytes/L; reference 1–5.4 × 10^9^/L).

Mean serum CRP levels did not differ between Group A (median = 10.6 mg/L; range 5.3–45.1) and Group B (median = 8.7 mg/L; range 6.5–13.5) (P = 0.08), nor between males and females (P = 0.63). Serum CRP was normally distributed only within Group B (mean CRP = 9.6 ± 2.3 mg/L). The fixed effect of CRP on CCECAI score was nearly significant at Pr > F = 0.06.

#### Association of endoscopic and histopathological findings with clinical signs and laboratory results

**Fig. 5:**
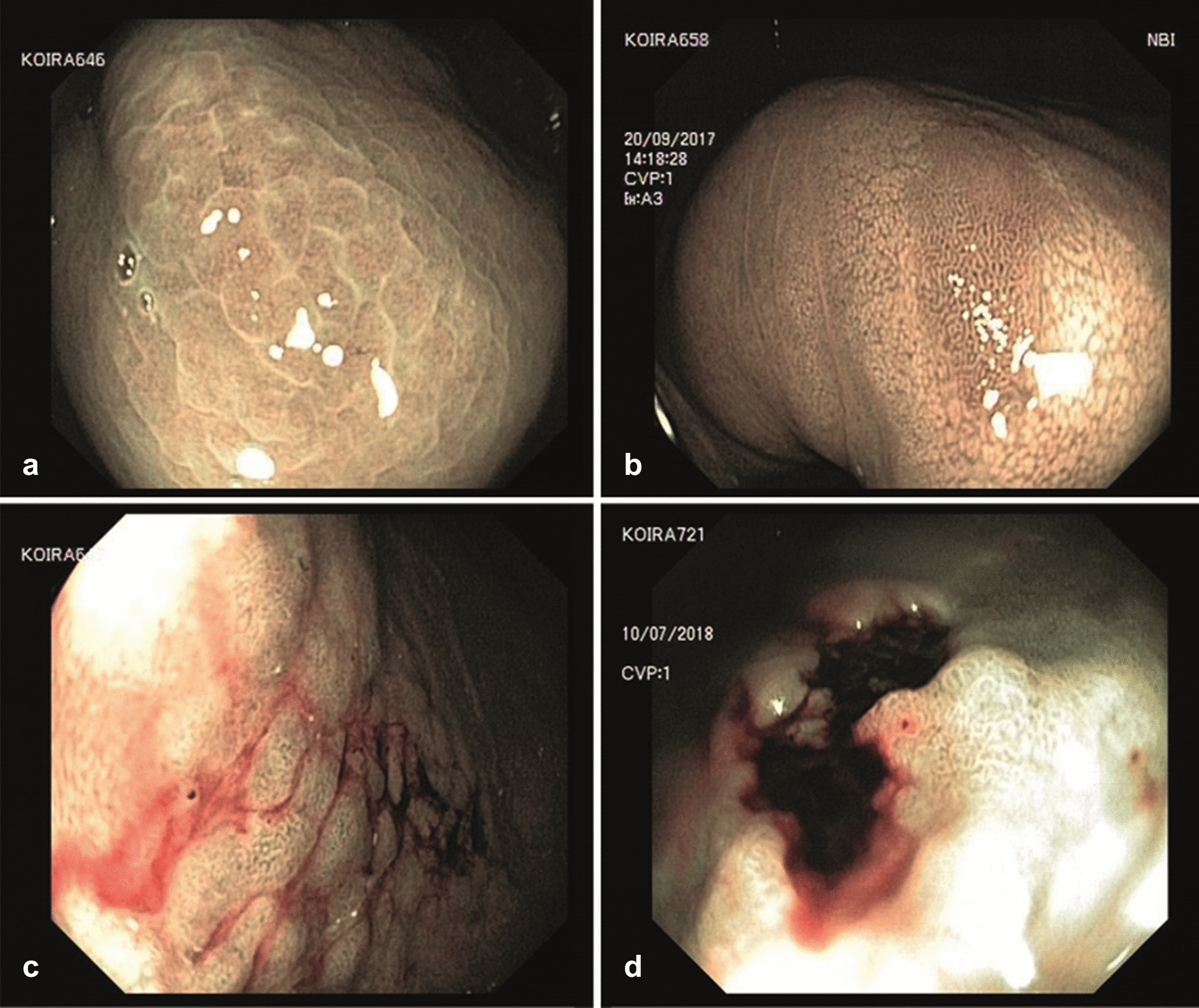
Endoscopic images showing examples of mucosal changes in the gastric body. **a** Mucous metaplasia (dog 646); **b** glandular dysplasia (dog 658), narrow band imaging (NBI); **c** early adenocarcinoma (dog 647); D: ulcerated carcinoma (dog 721; NBI)

At endoscopy, 5/27 dogs (one in Group A; four in Group B) had no distinct focal changes, hence the standard sampling protocol was applied (Fig. 1). Visible mucosal changes were present in 22/27 BSD: 21 dogs had changes affecting the gastric body and one dog had only minor focal changes restricted to the pyloric antrum. Endoscopic biopsy of both diffuse and focal mucosal changes supplied the respective histopathological diagnoses (Fig. 5).

The histopathological MSS did not differ significantly between Group A (1.6 ± 0.8) and Group B (1.3 ± 0.5) (P = 0.08), nor between males (1.2 ± 0.8) and females (1.6 ± 0.5) (P = 0.21). The MSS followed a normal distribution within both groups. Mean MSS was significantly higher in dogs vomiting once a month or more often (1.6 ± 0.7), than in those for which vomiting was reported as less frequent or absent (1.3 ± 0.6) (Pr > F = 0.028). Mean MSS tended to be higher in dogs diagnosed with glandular dysplasia (1.7 ± 0.6) than in those without dysplastic changes (1.2 ± 0.6) (Pr > F = 0.078). Moreover, an increased odds ratio for vomiting [OR = 9.9 (1.5–63.7)] was confirmed for dogs diagnosed with glandular dysplasia (P = 0.016) but not with mucosal atrophy, mucous metaplasia or GC.

Logistic regression analysis showed no statistical difference between Groups A and B concerning mucosal atrophy (A: 10/16; B: 10/11), mucous metaplasia (A: 4/16; B: 3/11), glandular dysplasia (A: 10/16; B: 3/11) nor GC (A: 4/16; B: 2/11). Regardless of grouping, most BSD had gastric mucosal atrophy (20/27), followed by glandular dysplasia (13/27), mucous metaplasia (6/27), and GC (6/27). Factors such as CCECAI score, GER and laboratory tests showed no significant associations with histopathology. Individual findings are summarized in Table 1.

Six dogs were diagnosed with GC (Tables 1, 2). All dogs with GC were over seven years of age (median = 9.2; range 7.2–11.2). GC concurred with changes elsewhere in the gastric mucosa in both groups, namely mucous metaplasia in 4/6, and mucosal dysplasia in all six dogs. Fisher’s exact test revealed a significant association of GC with mucous metaplasia (P = 0.026) and glandular dysplasia (P = 0.004), but not with mucosal atrophy (P = 1). All neoplasms were gastric adenocarcinomas (Table 2): 1/6 tubular type and 5/6 partly or completely of the diffuse, non-cohesive type (including one mucinous adenocarcinoma). In two patients (dog 711, Group A; dog 641, Group B), the endoscopic changes were more subtle, with no visible ulceration, nor bleeding after full insufflation. In the other four dogs, GC was diagnosed from ulcerated, bleeding masses.

**Table 2:** Breed type, sex, age and classification of gastric carcinomas diagnosed in Belgian Shepherd dogs with (Group A) or without upper gastrointestinal clinical signs (Group B)

Dog no	Breed type	Sex	Patient Group	Age at diagnosis (years)	Tumour classification [32, 33]
641	Tervuren	M	B	10.8	Tubular adenocarcinoma
647	Tervuren	F	A	10.1	Tubular with areas progressing to signet ring cell carcinoma
658	Tervuren	M	A	7.2	Mucinous adenocarcinoma
677	Groenendael	F	A	8.3	Signet ring cell carcinoma
711	Tervuren	F	A	8.3	Signet ring cell carcinoma
721	Groenendael	F	B	11.2	Tubular with areas progressing to signet ring cell carcinoma

Comparison of the CRP levels of all dogs in the study with histopathological findings including presence of GC (P = 0.44) failed significance. Notably, serum CRP of dog 686 was 45.1 mg/L, well over two standard deviations above the mean and therefore considered an outlier. When the subgroup of four dogs with ulcerated GC were compared to the other 20 dogs (i.e. except the abovementioned dogs 686, 711 and 641), the odds ratio for an increased CRP with ulcerated GC was nearly significant (P = 0.06), OR = 1.2 (0.99–1.48).

Neoplastic lesions were also accompanied by moderate (4/6) to severe (2/6) lymphoplasmacytic infiltration, and mild (1/6) to moderate (3/6, all non-cohesive GC) eosinophilic infiltration. The GC patient with the most severe clinical disease (CCECAI = 8) was both the youngest dog with GC (7.2 years) and the only one with a mucinous adenocarcinoma (dog 658).

**Fig. 6:**
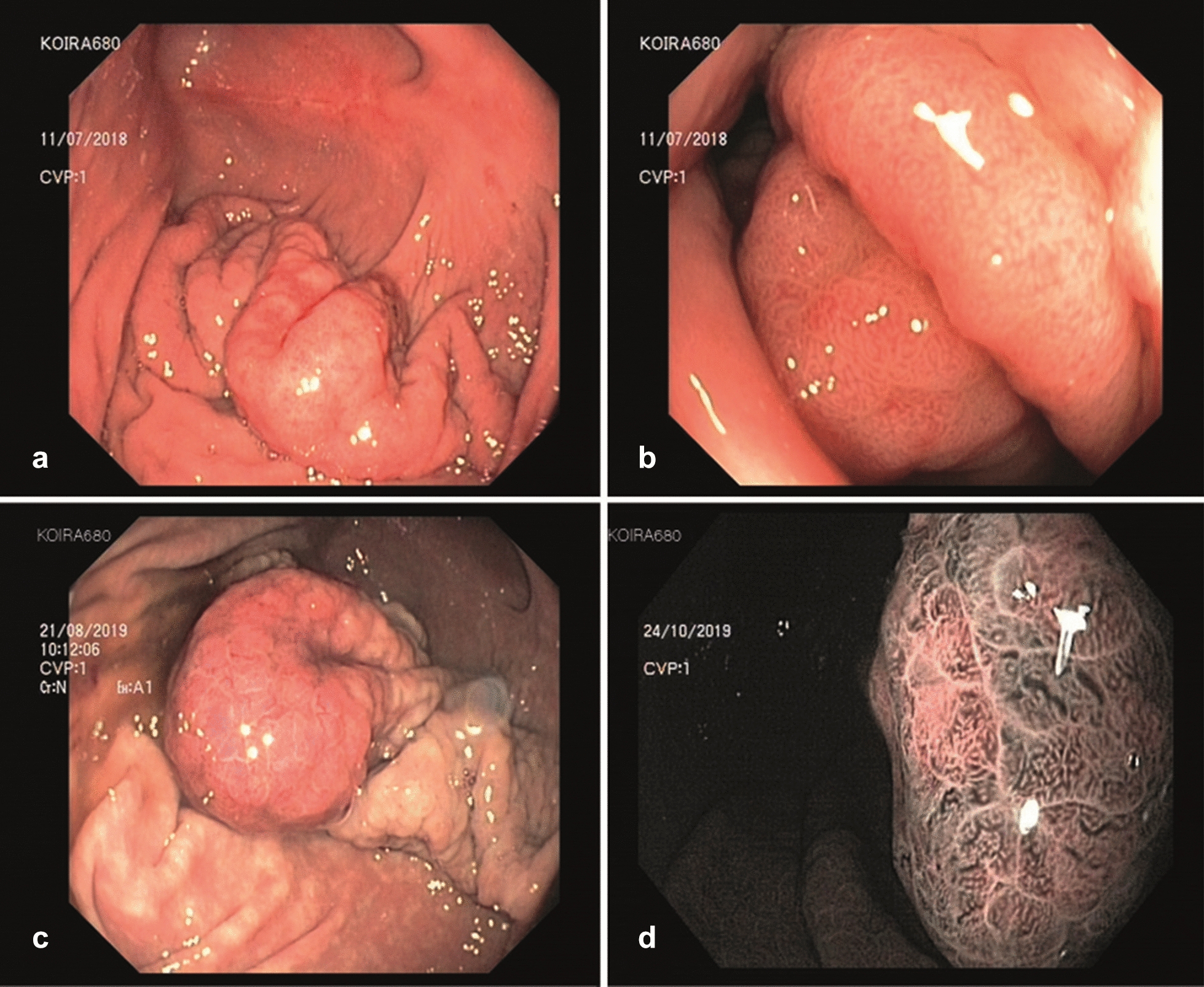
Dog 680. Overview and close-up endoscopic images of a focal mucosal change diagnosed as dysplastic both at initial examination (**a**, **b**) and one year later (**c**), i.e. after the study had ended. **a–c** White light endoscopy. **d** Narrow band imaging close-up just before surgical resection (confirmed tubular adenocarcinoma at histology)

In one case (dog 680; Group A), endoscopy revealed a marked fold of 4 × 7 cm in the distal greater curvature protruding into the lumen (Fig. 6). The change was soft, flexible and the mucosa lifted normally from the wall when taking biopsies (i.e. non-lifting sign absent) [35]. Its apex appeared superficially ulcerated and hyperaemic, with a disrupted blood vessel pattern in NBI. The most severe histological finding consisted of marked glandular dysplasia. At a voluntary follow-up endoscopy one year later, the change presented as more voluminous and clearly polypoid. The irregularity of the surrounding mucosa had grown in extension and coarseness, affecting most of the ventral gastric body towards the cardia. Surgery revealed no evidence of lymph node involvement and the polypoid mass was removed with a margin of 1.5 cm. Histopathology diagnosed a tubular adenocarcinoma with multifocal infiltration into the submucosa and clear margins. Four months later, no evidence for dysplastic nor neoplastic changes in the prominent edges of the resection scar was found with endoscopic biopsy: histopathology confirmed oedema, mild epithelial damage, lymphoplasmacytic gastritis and fibrosis. Biopsies from elsewhere in the gastric body showed mucous metaplasia. The patient had remained asymptomatic at the time of manuscript submission, a year after surgery.

## Discussion

The online survey revealed that BSD owners in Finland report a high percentage of clinical signs of gastric disease. Vomiting once a month or more often was reported in 43% of dogs. Vomiting was associated with poor appetite, gagging and changes in the dog´s attitude or behaviour, which is coherent with upper gastrointestinal malaise. The reported incidence of vomiting may suggest a relatively high prevalence of gastric illness in the BSD population. However, participation in the survey was voluntary. Owners were contacted in chronological order of response to minimize potential bias, but the possibility of volunteer bias [36], e.g. increased interest by those concerned about the outcomes of their individual dog’s examinations or impacts of the research, cannot be excluded. Therefore, conclusions were drawn with caution.

Eating grass was a common behaviour, reported in 87.5% of all dogs in the survey and in all but one of the patients undergoing endoscopy (dog 641, diagnosed with an early tubular adenocarcinoma). Eating grass failed to show significant association with vomiting. Plant-eating has been hypothesized to serve various purposes such as inducing vomiting or controlling gastrointestinal parasite burden [20, 23]. About 70% of all dogs eat plants on a daily or weekly basis, with grass being the most commonly consumed. However, very few dogs appear ill before (9%) or tend to vomit afterwards (22%) [22]. Eating grass has been regarded as normal behaviour in a controlled trial involving 12 mixed-breed dogs [24]. In our study, the actual frequency and amount of grass ingested were not recorded which to some degree limits the assessment of a possible association with gastric disease. Besides, eating foreign material also failed to show association with vomiting.

Vomiting was not applied as the single criterion for patient inclusion to Group A (with GI signs) or B (no GI signs). In the absence of a standardized, objective clinical measure of gastric disease, the Canine Chronic Enteropathy Clinical Activity Index was applied. The CCECAI is used to score signs such as inactivity, inappetence, weight loss and vomiting, which are recognized with upper gastrointestinal disorders, including GC [4, 18]. Nevertheless, a dog vomiting less often than once a week would score zero in CCECAI, whereas vomiting once a week would be regarded as mild, scoring one point. Subtle or initial illness might be overlooked in a patient with occult GC, presenting occasional vomiting as the only clinical sign. To avoid this drawback, a wider range of clinical signs which are often associated with GER were used for inclusion to Group A. Thus, the dogs in Group B could be clearly considered asymptomatic in regard to GI signs.

WBC below the reference range occurred in dogs of both groups (7/27; 25.9%), with and without GC. Low WBC in itself does not entail an increased risk for anaesthesia or endoscopic procedures and was therefore not regarded as a reason for exclusion. Leukopenia has been proposed as common and possibly even physiological for Belgian Tervuren in a study in North America [37], but the condition was unconfirmed in European lineages: Gommeren et al. [38] found leukopenia in only 1/94 healthy Belgian Tervuren, although WBC significantly decreased with patient age. Our study in BSD older than five years revealed neither American progenitors in the pedigree of dogs with low WBC, nor an association of age with WBC. A previous retrospective study [17] reported lymphopenia in 44% and leucocytosis in 21% of dogs of various breeds with gastric histopathological abnormalities. Nevertheless, low WBC showed no association with either clinical or histopathological variables in BSD in this study.

Serum CRP has been reported as increased in dogs diagnosed with GC as compared to dogs with chronic gastritis and control dogs [18]. In humans with GC, CRP shows limited evidence as a single prognostic marker [39]. Our study found no difference in CRP between Groups A and B, nor when the six GC patients were compared with all other dogs (Table 1). However, when the subgroup of dogs with ulcerated GC was compared with the other 20 dogs (except two non-ulcerative GC and one outlying CRP result), logistic regression pointed towards a possible association of CRP with ulcerated GC (P = 0.06). The analysis is underpowered as it draws on the comparison of only four dogs with visibly ulcerated GC and 20 dogs without GC. Measuring CRP in stored samples might be erroneous, but studies reassure that, in human serum frozen at − 20 °C, CRP remains stable for at least 1026 days [40].

Previous reports have shown higher incidence of GC in male dogs [1]. Our online survey results suggested that male BSD are approximately twice as likely to vomit as females. However, our relatively small sample size in the endoscopic study prevents assessing sex as a factor for GC (four of the six dogs with GC were females). Moreover, no statistic difference was found between males and females concerning CCECAI score, CRP, WBC, or the MSS of gastric biopsies.

None of the BSD in our study had intestinal metaplasia diagnosed from endoscopic biopsies. This suggests that, at least in this population, the histological pathway towards GC does not follow the human linear cascade proposed by Correa [44]. The most common gastric tumour in man, arising via that model, is the intestinal-type gastric adenocarcinoma [11]. In our BSD study population, however, different types of carcinoma were described: 1/6 intestinal/tubular type and 5/6 presenting traits of the diffuse, non-cohesive type (including one mucinous adenocarcinoma). These findings in BSD further illustrate possibly divergent pathogenetic pathways in canine GC [4].

Extensive efforts to standardize inflammatory changes in canine gastrointestinal pathology have contributed to diagnostic capabilities in this field [31, 34, 41]. However, the occurrence and role of possible preneoplastic mucosal changes in dogs has not been as thoroughly studied and it remains unproven whether carcinogenic mechanisms established in humans are relevant in dogs. In man, the classic pathway of carcinogenesis is ascribed to chronic, atrophic gastritis followed by intestinal metaplasia and dysplasia, which are recognized as preneoplastic changes [44]. Regardless of clinical signs, or severity of histopathological changes, 74% of our dogs had gastric mucosal atrophy and it carried no statistical association with GC. In veterinary medicine, only gastric mucous metaplasia has been mentioned as potentially preneoplastic [33]. Out of the six BSD with GC, five also had mucosal atrophy, four had mucous metaplasia, and all six had dysplastic changes as well as moderate to severe chronic inflammation, including eosinophilic infiltration, affecting mainly the gastric body. This differs from the neuroendocrine carcinomas described in four of eight Norwegian Lundehund with gastric neoplasia [42], wherein only mild antral inflammation has been reported [43].

Relevant histopathological changes, including GC, were diagnosed in both groups A and B. Our approach to group allocation involved an array of gastrointestinal signs, which were analysed as presence/absence of signs of GER and individual scores for CCECAI. Ultimately, vomiting was the only sign showing an important correlation with MSS and notably with glandular dysplasia (OR = 9.9). There was a trend for increased MSS along with glandular dysplasia (Pr > F = 0.078, close to significance). That may suggest a possible pathophysiological association of chronic gastritis with carcinogenesis also in BSD, warranting studies with higher statistical power. Both mucous metaplasia and especially glandular dysplasia correlated with GC, which is also the case in the classic human model of gastric carcinogenesis [4, 44].

Vomiting is regarded as the cardinal sign for gastric localization and consistently mentioned in previous studies on canine GC [4, 7–10, 18]. Nevertheless, vomiting was mild or absent in some GC cases, and it showed no correlation with mucosal atrophy, mucous metaplasia, nor GC. Moreover, 6/27 (22%) BSD over 5.5 years of age were diagnosed with GC regardless of clinical condition. This further corroborates a marked predisposition to GC in these ageing Tervuren and Groenendael BSD, as GC is rare in dogs, except for a few predisposed breeds [1, 6]. Even more importantly, it shows that gastric pathology including GC can occur as an occult disease in affected dogs. Such evidence supports a clinical indication for the procurement of gastric biopsies in ageing BSD, before the lesions become locally advanced or metastasise, carrying a poor prognosis.

Early detection of GC can potentially improve patient outcome, enabling surgical removal before such tumours have developed in size and clinical severity, as illustrated by the follow-up of dog 680 (group B) as well as another study showing prolonged survival time for a few dogs treated having only minimally invasive GC [9]. Timely diagnosis may facilitate potential surgical or minimally invasive treatment and avoid unnecessary suffering, as well as contribute to the understanding of GC pathophysiology in dogs. In order to clarify whether mucous metaplasia and glandular dysplasia of canine gastric mucosa are preneoplastic changes, as already established in human medicine, future clinical studies should involve endoscopic surveillance of affected dogs. For such studies, recording the exact localization of lesions in a schematic gastric map could be useful to investigate the development of gastric pathology in subsequent endoscopies [30].

Alongside non-predisposed breeds, a great challenge remains in diagnosing the asymptomatic GC cases. Extensive endoscopic screening of healthy BSD to diagnose occult GC may be unwarranted and thus ethically questionable. As opposed to veterinary medicine, several standard laboratory tests and tumour markers are useful in screening and prognosing human GC [45–52]. As shown in our study, CRP correlates well with prognosis, but as a non-specific inflammatory protein it shows limited level of evidence and potential as an early marker [53]. Therefore, future research aiming at early diagnosis of GC in dogs should also focus on non-invasive serum or faecal biomarkers [18].

## Conclusions

This study on ageing Tervuren and Groenendael BSD showed a high proportion of dogs with clinical signs of gastric disease regardless of the presence of GC. Gastric mucosal pathology included atrophy, mucous metaplasia, glandular dysplasia and GC, which were found irrespective of clinical signs.

Recurrent vomiting correlated with the severity of mucosal inflammation and presence of dysplasia and should be regarded as an indication for endoscopy and gastric mucosal sampling in BSD. Moreover, the significant association of GC with metaplasia and dysplasia, which are regarded as premalignant in humans, supports the indication for endoscopic surveillance in BSD. However, extensive endoscopic screening due to the occurrence of occult gastric disease remains unrealistic. Therefore, sensitive and specific indicators of GC such as serum or faecal biomarkers are warranted to promote early diagnosis of canine GC.

## Data Availability

The datasets used and/or analysed during the current study are available from the corresponding author on reasonable request. The original questionnaire in Finnish responded by the participating dog owners is available at: https://elomake.helsinki.fi/lomakkeet/75242/lomake.html. The same questionnaire is found in English at: https://elomake.helsinki.fi/lomakkeet/77393/lomake.html.

## Abbreviations

BSD: Belgian Shepherd dogs
CBC: Complete blood count
CCECAI: Canine chronic enteropathy clinical activity index
CRP: C-reactive protein
GC: Gastric carcinoma
GER: Gastroesophageal reflux
GI: Gastrointestinal
HER: Human epidermal growth factor receptor-type 2
MSS: Mean severity score for mucosal inflammatory infiltration
NBI: Narrow band imaging
OR: Odds ratio
RR: Risk ratio
WBC: Total leucocyte count
WLE: White light endoscopy
